# Exploring the therapeutic mechanisms of heart failure with Chinese herbal medicine: a focus on miRNA-mediated regulation

**DOI:** 10.3389/fphar.2024.1475975

**Published:** 2024-11-05

**Authors:** Yang Wang, Junyu Lai, Zhengtao Chen, Liqiang Sun, Yirong Ma, Jianguang Wu

**Affiliations:** ^1^ Department of Postgraduate, Jiangxi University of Chinese Medicine, Nanchang, China; ^2^ Department of Cardiovascular, Affiliated Hospital of Jiangxi University of Chinese Medicine, Nanchang, Jiangxi, China

**Keywords:** Chinese herbal medicine, microRNA, heart failure, therapeutic strategies, cardiac hypertrophy, cardiac fibrosis, apoptosis

## Abstract

Heart failure (HF) is a clinical condition caused by abnormalities in the heart’s structure or function, primarily manifested as diminished ability of the heart to pump blood, which leads to compensatory activation of neurohormones and increased left ventricular filling pressure. HF is one of the fastest-growing cardiovascular diseases globally in terms of incidence and mortality, negatively impacting patients’ quality of life and imposing significant medical and economic burdens. Despite advancements in the treatment of HF, hospitalization and mortality remain rates high. In China, Chinese herbal medicine (CHM) has historically played a prominent role in addressing HF, with significant proven efficacy. MicroRNA (miRNA) exerts a pivotal regulatory influence on the maintenance of regular cardiac activity and the progression of HF. MiRNAs, a category of single-stranded RNA molecules, are characterized by their inability to code for proteins. They regulate gene expression by binding to the 3′-untranslated region (3′-UTR) of target mRNAs, thereby influencing the onset and progression of various diseases. Abnormal expression of specific miRNAs is closely associated with HF pathological processes, such as cardiomyocyte apoptosis, myocardial fibrosis, and cardiac hypertrophy. This abnormal expression can influence the pathological progression of HF through the regulation of miRNA expression. This article reviews the regulatory role of miRNAs in HF pathology discusses how CHM compounds and their active ingredients can ameliorate HF pathology through the regulation of miRNA expression. In conclusion, miRNAs represent promising therapeutic targets for HF, and CHM provides a novel strategy for treatment through the regulation of miRNA expression. Future studies must delve deeper into the precise mechanisms by which CHM modulates miRNAs and fully explore its potential for clinical application in HF treatment.

## 1 Introduction

Heart failure (HF) is a clinical condition resulting from structural or functional impairments of the heart (Amgalan and Kitsis, 2019). Traditionally, HF is characterized by a decrease in the heart’s pumping and/or congestion ability, stemming from abnormal cardiac structure or function that results in insufficient cardiac output, leading to compensatory neurohormonal activation and increased left ventricular filling pressure (Savarese et al., 2023). HF frequently occurs in the terminal stages of various organic heart ailments and other diseases, making it one of the fastest-growing cardiovascular diseases globally in terms of incidence and mortality rates. Over the past few decades, despite considerable progress in the management of HF, hospitalization rates and mortality have remained stubbornly high, making HF a significant factor that severely impacts patients’ quality of life. Generally, the best treatment for HF is involves an individualized plan, which may include general treatment, drug therapy, surgical interventions, and other approaches aimed at alleviating physical discomfort. However, the prognosis for HF after treatment is still poor, and the recent hospitalization rate and related medical expenses of HF following treatment remains poor, and recent hospitalization rates and associated medical expenses have increased significantly (Iyngkaran et al., 2018; Ziaeian and Fonarow, 2016). In 2012, total expenditure on HF treatment in the United States amounted $30.7 billion. Forecasts indicate that by 2030, this amount is projected to escalate to an alarming $69.7 billion (Sinnenberg and Givertz, 2020). Furthermore, the absolute number of HF hospitalizations is also projected to increase dramatically over the next 25 years, potentially by as much as 50% (Al-Mohammad et al., 2010; Savarese and Lund, 2017). Therefore, it is imperative that we to comprehend the etiology of HF while concurrently innovating novel therapeutic approaches and more effective medications to combat this escalating health crisis.

Currently, the primary treatment for HF involves modern biomedicine; however, conventional treatment targets of modern biomedicine are relatively limited, lacking distinctive personalized treatment features and associated with numerous adverse drug reactions (Jia et al., 2020). Traditional Chinese Medicine (TCM), a unique medical system in China with a long history, is widely practiced in folklore and applied in the treatment and conditioning of various diseases. TCM not only demonstrates significant therapeutic effects but also exhibits fewer side effects (Fan et al., 2022) Furthermore, TCM, especially Chinese herbal medicine (CHM), holds unique advantages in regulating the body, preventing diseases, and promoting overall health. Consequently, TCM occupies a significant position within the modern medical system (Li et al., 2020). Recent studies have highlighted the positive effects of CHM active components, including tanshinone IIA (Tan IIA), *Lycium barbarum* polysaccharide (LBP), ginsenosides, *Panax notoginseng* saponins (PNS), and Total glucosides of paeony (TGP) on the cardiovascular system. These components exhibit anti-apoptotic, antioxidant, anti-inflammatory, and antifibrotic effects, providing substantial therapeutic benefits for HF (Hao et al., 2017). The multi-channel and multi-target advantages of TCM enable it to circumvent issues associated with modern biomedicine, such as adverse effects arising from genetic defects in therapeutic targets and diminished drug efficacy due to metabolic genetic variations (Wang et al., 2017). Therefore, CHM and its active ingredients exhibit considerable potential for application in the treatment of HF. Currently, a growing body of research is focused on the therapeutic effects of CHM on HF, aiming to elucidate its underlying mechanisms and explore its applications in modern medicine. Through systematic scientific research and clinical trials, CHM has the potential to become a significant supplement and a key direction for development in HF treatment.

**FIGURE 1:**
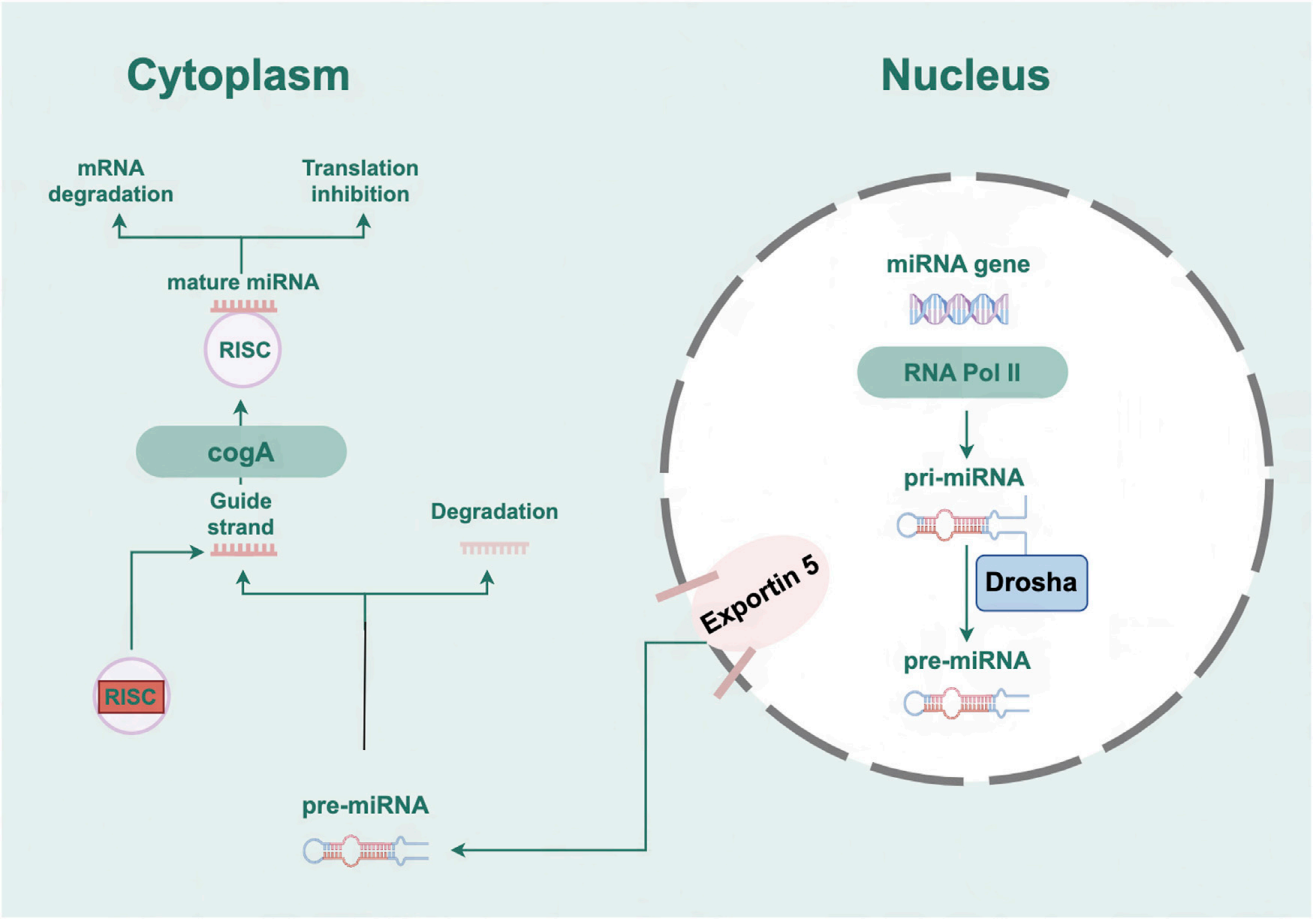
The biogenesis and mechanism of action of microRNAs.

MicroRNAs (miRNAs), characterized by their single-stranded RNA structure, comprise 19 to 25 nucleotides and are incapable of encoding proteins. By binding to the 3′-UTR, these molecules are known to suppress the post-transcriptional expression of mRNA (Liu et al., 2019). The process of miRNA biogenesis is initiated by the transcription of primary miRNA transcripts, known as pri-miRNAs. These pri-miRNAs are subsequently subjected to a series of modifications, transforming them into precursor miRNAs, known as pre-miRNAs. Following a cascade of events, the pre-miRNAs mature, resulting in functional miRNAs that integrate into the RNA-induced silencing complex (RISC) to execute their regulatory roles (Treiber et al., 2019) (Figure 1). MiRNAs are pivotal regulatory elements in post-transcriptional processes and have emerged as a novel category of gene expression modulators integral to both human health and the development of pathologies (Michlewski and Cáceres, 2019; Sucharov et al., 2017). Growing evidence suggests that miRNAs occupy a pivotal position in regulating various aspects of cardiac function and the progression of HF (Zhang R. et al., 2018). Therefore, enhancing the understanding of miRNAs’ roles could contribute to uncovering novel mechanisms, identifying biomarkers, and pinpointing potential targets for cardiovascular conditions.

Within cardiac tissue, miRNAs serve as regulators, impacting vital processes such as heart development, fibrosis progression, inflammatory responses, and tissue regeneration (Thum and Condorelli, 2015). The upregulation or downregulation of specific miRNAs can directly affect the function and structure of cardiomyocytes, thereby facilitating the progression of HF (Gargiulo et al., 2023). Currently, a multitude of research efforts are concentrating on the link between miRNAs and the application of CHM in treating HF. It is particularly noteworthy that CHM has demonstrated therapeutic effect on HF by regulating miRNA expression (Wu et al., 2023). These studies indicate that miRNAs have broad potential applications in the future practice of CHM of HF treatment and are expected to become key therapeutic intervention targets.

This article delves into the pivotal functions of miRNAs in processes such as myocardial hypertrophy, fibrosis, and apoptosis, as well as the specific mechanisms by which CHM regulates miRNAs. To this end, literature searches on HF were conducted using databases such as Web of Science, PubMed, ScienceDirect, Springer, Wiley, focusing on studies published between 2013 and 2023. The keywords “heart failure” and/or “miRNA” were employed, either individually or in combination with terms such as “CHM”, “fibrosis”, “autophagy”, “apoptosis”. The research findings from these searches are presented as follows.

## 2 MiRNAs participate in the progression of HF

**FIGURE 2:**
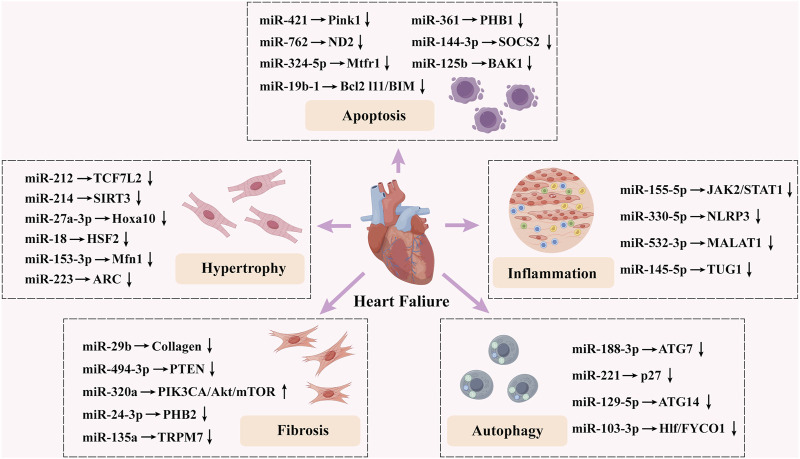
Summary of the impact of microRNAs on heart failure.

The emergence and progression of HF are complex, involving mechanisms such as apoptosis, autophagy of cardiac myocytes, cardiac myocyte hypertrophy, cardiac fibrosis, and inflammation (Triposkiadis et al., 2022). However, miRNAs are involved in nearly all of these pathological processes associated with HF and play a crucial regulatory role (Figure 2).

### 2.1 MiRNAs regulate myocardial cell apoptosis

Apoptosis of myocardial cells represents a crucial pathophysiological mechanism in HF and serves as a key driver in its progression (Xue et al., 2023). Apoptosis is a death process caused by a sequence of cellular death process resulting from a series of changes in response to environmental signals, altered environmental conditions, or compensatory injuries, playing a crucial role in the clearance of infected cells (Corsetti et al., 2019). Since mitochondria are the primary organelles responsible for reactive oxygen species and ATP within cardiomyocytes, disruptions in mitochondrial energy supply, cell death, and dysfunction play a critical role in the pathogenesis of HF (Chen et al., 2011). Increasingly, studies have demonstrated that miRNAs are involved in regulating mitochondrial morphology in cardiomyocytes, and in the process of endoplasmic reticulum stress-induced injury and apoptosis (Zhou et al., 2023). This section primarily discusses the impact of miRNA on mitochondrial functionality and the stress experienced by the endoplasmic reticulum in cardiomyocytes during HF, elucidating their protective role against apoptosis and potential therapeutic strategies (Table 1).

**TABLE 1:** Mechanism of action of miRNAs involved in HF progression.

	miRNA	Target	Function	Reference
Apoptosis	miR-421	Pink1	Promoting mitochondrial fragmentation and apoptosis in cardiomyocytes	Wang et al. (2015d)
	miR-762	ND2	Regulate mitochondrial function and induce apoptosis	Yan et al. (2019)
	miR-324–5p	Mtfr1	Alleviate mitochondrial fission and apoptosis	Wang et al. (2015c)
	miR-361	PHB1	Trigger mitochondrial fission and apoptosis	Wang et al. (2015a)
	miR-144–3p	SOCS2	Alleviates DOX induced apoptosis and cardiac dysfunction	Zhang et al. (2023a)
	miR-125b	BAK1	Inhibition of cardiomyocyte apoptosis	Zhang et al. (2021)
	miR-19b-1	Bcl2 l11/BIM	Inhibition of cardiomyocyte apoptosis	Yang et al. (2019)
Autophagy	miR-188–3p	ATG7	Inhibition of autophagy and apoptosis	Wang et al. (2015b)
	miR-221	p27	Influencing cardiac remodeling and autophagy	Su et al. (2015)
	miR-129–5p	ATG14	Inhibition of autophagy and apoptosis	Zhang et al. (2018a)
	miR-103–3p	Hlf/FYCO1	Inhibition of autophagy and apoptosis	Xue et al. (2023)
Fibrosis	miR-29b	Collagen	Inhibit the progression of cardiac fibrosis	Dawson et al. (2013)
	miR-494–3p	PTEN	Promote myocardial fibrosis	Tang et al. (2023)
	miR-320a	PIK3CA/Akt/mTOR	Promote the growth of cardiac fibroblasts	Wang et al. (2021b)
	miR-24–3p	PHB2	Alleviate cardiac fibrosis	Zhang et al. (2022b)
	miR-135a	TRPM7	Inhibition of ISOP-induced myocardial fibrosis	Wu et al. (2018)
Hypertrophy	miR-212	TCF7L2	Induction of cardiomyocyte hypertrophy	Yuan and Yuan (2022)
	miR-214	SIRT3	Induced cardiomyocyte hypertrophy	Ding et al. (2021)
	miR-27a-3p	Hoxa10	Alleviate cardiac hypertrophy	Cao et al. (2021)
	miR-153–3p	Mfn1	Promotes mitochondrial division and cardiomyocyte hypertrophy	Wang et al. (2020)
	miR-18	HSF2	Protect cardiomyocytes from hypertrophy	Huang et al. (2017)
	miR-223	ARC	Protecting the myocardium from pathologic hypertrophy and heart failure	Wang et al. (2016)
Inflammation	miR-155–5p	JAK2/STAT1	Enhance the inflammatory response	Ge et al. (2021)
	miR-330–5p	NLRP3	Alleviate myocardial I/R injury and inflammation	Zuo et al. (2021)
	miR-532–3p	MALAT1	Reduces myocardial cell damage and inflammation	Zhao et al. (2021)
	miR-145–5p	TUG1	Alleviate hypoxia-induced inflammation in cardiomyocytes	Zhu et al. (2021)

Wang et al. (2015d) discovered that miR-421 is involved in suppressing the translation of Pink1, leading to myocardial infarction, apoptosis, and mitochondrial fragmentation by decreasing Pink1 expression, a serine/threonine kinase that targets mitochondria and inhibits cardiomyocyte apoptosis (Siddall et al., 2013). Moreover, E2F1 is a key member of the E2F family of transcription factors, essential for cardiac function and primarily influencing cardiomyocyte metabolism (Dassanayaka et al., 2019). E2F1 can transcriptionally activate the expression of miR-421, thereby regulating mitochondrial fission and promoting apoptosis. Knockdown of E2F1 can suppress mitochondrial fission and apoptosis in myocardial cells. These findings establish the E2F1/miR-421/Pink1 signaling pathway as a critical regulatory factor for mitochondrial division and cell death in myocardial cells. Additional research indicates that miR-762 plays a crucial role in modulating mitochondrial function and inducing cardiomyocyte apoptosis via the core assembly subunit ND2 of mitochondrial complex I. As a novel contributor to mitochondrial dysfunction, miR-762 may also serve as an effective therapeutic target for HF (Yan et al., 2019). Wang et al. (2015c) found that miR-324–5p acts as a suppressor of translation for the mitochondrial fission regulator 1 (Mtfr1). They further elucidated that this miRNA mitigates mitochondrial fission, apoptosis, and myocardial infarction by decreasing expression levels of Mtfr1. However, NFAT4, a gene located on mouse chromosome 8, exerts a suppressive influence on miR-324–5p. This gene is pivotal in reducing voltage-gated potassium currents following myocardial infarction. Moreover, its regulatory role extends to influencing mitochondrial fission and the initiation of apoptosis. This study establishes the NFAT4/miR-324–5p/Mtfr1 pathway as involved in the modulation of mitochondrial division and programmed cell death in myocardial cells, offering a potential new therapeutic approach for heart disease. Similarly, another study found that miR-361 is involved in inhibiting the translation of prohibitin 1 (PHB1). MiR-361 triggers mitochondrial fission, apoptosis, and HF by reducing PHB1 levels. Knockdown of miR-361 increases PHB1 levels and reduces mitochondrial fragmentation and apoptosis both *in vivo* and *in vitro*, thereby protecting the heart from ischemic injury (Wang et al., 2015a). The research reveals a novel mechanism for regulating mitochondrial fragmentation and apoptosis involving miR-361 and PHB1. This finding may provide novel insights into therapeutic approaches for myocardial infarction and HF.

Additionally, some miRNAs are involved in regulating myocardial cell apoptosis through alternative pathways or signaling mechanisms. Zhang D. et al. (2023) discovered that increased levels of miR-144–3p mitigates HF and cardiomyocyte apoptosis induced by doxorubicin (Dox) in rats, acting through the SOCS2/PI3K/AKT signaling pathway. In Dox-treated cardiomyocytes, suppression of SOCS2 counteracted the downregulating effects of miR-144–3p. The upregulation of miR-144–3p reduced apoptosis and heart dysfunction caused by Dox by targeting SOCS2. This finding provides new insights into the role of miR-144–3p in HF. In another study, researchers observed a significant reduction in miR-125b levels in the cardiac muscle of mice afflicted by HF. Overexpression of miR-125b can inhibit myocardial cell apoptosis by targeting the Bcl-2 homologous antagonist/killer (BAK1), effectively alleviating cardiac dysfunction in HF mice. This suggests that targeting the miR-125b/BAK1 pathway may be a viable strategy for the detection or management of HF (Zhang et al., 2021). Yang et al. (2019) found that miR-19b-1, a key component of the miR-17/92 cluster, is essential for curbing cell death. miR-19b-1 targets the pro-apoptotic Bcl-2 family gene (Bcl2l11/BIM), reducing its mRNA and protein levels, thereby reversing ischemia-induced HF by inhibiting myocardial cell apoptosis.

### 2.2 MiRNAs regulate autophagy in myocardial cells

Autophagy is a process in which impaired organelles and abnormal protein aggregates, among other large cellular components, are enclosed within lysosomes for subsequent degradation. This process is crucial for maintaining cell survival, differentiation, development, and homeostasis (Mizushima and Levine, 2010). Autophagy is intimately linked to the maintenance of cardiac equilibrium, both under healthy conditions and in pathological states such as myocardial hypertrophy, cardiac fibrosis, myocardial infarction, HF, and other structural heart diseases (Lampert and Gustafsson Å, 2018). Under stress stimuli, autophagy can function both as a protective mechanism that promote the survival of myocardial cells and as a maladaptive process that induces the death (Delbridge et al., 2017). Additionally, research indicates that miRNAs serve as pivotal regulators of the autophagy process, exerting significant influence over its modulation in cardiomyocytes through both transcriptional and post-transcriptional regulatory mechanisms. This section focuses on how autophagy and associated miRNAs participate in and influence the initiation and advancement progression of HF, offering a new perspective for exploring therapeutic strategies for HF.

Autophagy involves a suite of conserved *Atg* gene sequences that are present from yeast to humans. ATG7 is recognized as a significant biomarker for autophagy and also functions as an essential initiator of the autophagic process, playing key regulatory roles in cell death and survival (Pattison et al., 2011). In both *in vivo* and *in vitro* studies, Wang et al. (2015b) found that miR-188–3p specifically targets ATG7, thus inhibiting autophagy and apoptosis. However, research has demonstrated that an autophagy-promoting factor (APF) can directly interact with miR-188–3p, thereby dampening its activity and modulating autophagy and autophagic cell death through the miR-188–3p/ATG7 signaling pathway. This study reveals a novel miRNA-related model for regulating autophagy programs, with the potential for level modulation to be utilized as a diagnostic indicator and target for developing innovative treatments for HF. Su et al. (2015) discovered that in mice, overexpression of miR-221 specifically in the heart induced cardiac dysfunction and HF, attributed to the suppression of autophagy by miR-221. Specifically, p27, a cyclin-dependent kinase (CDK) inhibitor, is identified as a primary molecular target of miR-221 in cardiomyocytes. In cardiomyocytes, miR-221 orchestrates the p27/CDK2/mTOR signaling pathway, thereby influencing cardiac remodeling and autophagy in the heart. which is a classic pathway exerting negative regulation on autophagy (Zhang H. et al., 2018). miR-129–5p has been confirmed to suppress autophagy and apoptosis triggered by H2O2 in rat cardiomyocytes (H9C2) by lowering ATG14 levels and activating the PI3K/Akt/mTOR pathway (Zhang et al., 2020). Mi et al. (2023) found that the maternal expression of gene 3 (MEG3) mediates HF and excessive cardiac remodeling in mice induced by subcutaneous injections of isoproterenol (ISOP). The reduction of MEG3 levels notably inhibits the excessive apoptosis and autophagy in cardiomyocytes provoked by ISOP and H2O2. This effect is achieved by modulating the miRNA-129–5p/ATG14/Akt signaling pathway. Xue et al. (2023) found that the hepatic leukemia factor Hlf/FYVE and the protein containing coiled-coil domain 1 (FYCO1) are prospective targets for miR-103–3p. This miRNA exerts its effects on apoptosis and autophagy through repressing Hlf expression via binding to the 3′-UTR region of the Hlf/FYCO1 gene.

### 2.3 MiRNAs regulate myocardial cell fibrosis

Myocardial fibrosis is associated with an unfavorable prognosis in heart-related conditions and is one of the most prevalent pathophysiological outcomes triggered by stimuli that can be either acute, such as myocardial infarction, or chronic, such as hypertension (Hinderer and Schenke-Layland, 2019; Xing et al., 2024). In the myocardium, cardiac fibrosis manifests as a scarring process, characterized by the proliferation and transformation of cardiac fibroblasts into myofibroblasts influenced by mechanical stimuli, pressure/volume overload, and circulating humoral factors. This leads to an overproduction of extracellular matrix (ECM), a reduction in the heart’s adaptability, and initiates structural transformation, culminating in HF (Gourdie et al., 2016; Travers et al., 2016). Furthermore, myocardial fibrosis correlates with both ventricular dysfunction and arrhythmias, serving as a negative predictor of outcomes for individuals with HF (Frangogiannis, 2019).

Recent findings indicate that the miR-29 family regulates cardiac metabolism across various cardiovascular tissues, including aortic tissues, myocardial tissues, vascular endothelial cells, and cardiomyocytes(Liu et al., 2021). The miR-29 family, specifically miR-29a and miR-29b, shows promise as potential predictive biomarkers for the progression of HF (Lakhani et al., 2018). In a canine model of CHF, the expression of miR-29b was reduced, accompanied by a marked increase in collagen I/III and fibrillin levels in extracted myocardial fibroblasts. Knocking down miR-29b in canine atrial fibroblasts using a lentivirus resulted in an increase in collagen expression. Conversely, elevated levels of miR-29b correlated with reduced collagen levels (Dawson et al., 2013). Activation of AMPK suppresses hepatocyte nuclear factor 4α (HNF-4α) transcription factor expression. This suppression leads to decrease TGF-β1 levels and increased miR-29 expression. These coordinated effects ultimately inhibit the progression of cardiac fibrosis and improve heart function in rats affected by this condition (Qi et al., 2017). Peli1, recognized as an E3 ubiquitin ligase, plays a broad role in signaling cascades mediated by tumor necrosis factor receptors (TNFRs) and Toll-like receptors (TLRs)/IL-1receptors (IL-1Rs) (Medvedev et al., 2015). Silencing Peli1 specifically in cardiomyocytes (CMs) can improve left ventricular fibrosis and cardiac dysfunction following myocardial infarction (Wu et al., 2014). Tang et al. (2023) discovered that Peli1 promotes myocardial fibrosis through cardiomyocyte-derived exosomes enriched with miR-494–3p in the HF model induced by pressure overload, offering a potential exosome miRNA-based treatment for cardiac fibrosis. In another exosome miRNA study. Wang Q. G. et al. (2021) identified a significant correlation between clinical markers of CHF and serum miR-320a levels. In contrast to exosomes derived from healthy individuals, exosomes extracted from patients with CHF exhibited a marked elevation in miR-320a expression. Subsequently, embryonic fibroblast-like cells (HEH2) were treated with miR-320a mimics and inhibitors. The investigation revealed that miR-320a from serum exosomes could enhance the growth of cardiac fibroblasts via the PIK3CA/Akt/mTOR signaling pathway in HEH2 cells. This finding suggests that miR-320a within serum exosomes may serve as a promising diagnostic indicator for CHF.

MiR-24–3p functions as a tumor suppressor and is involved in various cancer processes (He et al., 2020; Ye et al., 2016). Additionally, miR-24–3p reduces I/R-induced cardiomyocyte apoptosis and is associated with collagen synthesis in cardiac fibrosis (Yang et al., 2022). Prohibitin 2 (PHB2), an autophagy receptor located on the inner mitochondrial membrane, has been identified as a direct target of miR-24–3p. This miRNA mitigates cardiac fibrosis by suppressing autophagy in cardiac fibroblasts through the downregulation of PHB2 (Zhang et al., 2022b). Reduced levels of miR-135a correlate with the proliferation and invasiveness of cancer cells (Dang et al., 2014). This miRNA is also implicated in regulating the expression of the sodium-calcium exchanger, thereby influencing cardiac electrical activity (Duong et al., 2017). MiR-135a also demonstrate a potential link to cardiac fibrosis. *In vitro* experiments show that miR-135a expression is significantly reduced after ISOP treatment of neonatal rat cardiac fibroblasts. Furthermore, miR-135a mimics inhibit cardiac fibroblast proliferation and differentiation by suppressing transient receptor potential melastatin 7 (TRPM7) expression and its associated currents *In vivo* experiments indicate that, after ISOP-induced cardiac fibrosis in adult SD rats, TRPM7 expression is upregulated, while miR-135a expression is downregulated in cardiac tissue (Wu et al., 2018). miR-135a inhibits ISOP-induced cardiac fibrosis by regulating the TRPM7-mediated collagen production pathway, indicating its protective role in modulating cardiac fibrosis.

### 2.4 MiRNAs induce cardiomyocyte hypertrophy

Pathological cardiac hypertrophy represents the heart’s adaptive mechanism in response to increased stress, which can arise from various cardiovascular afflictions, including valvular heart disease, hypertension, and myocardial infarction. Although initially beneficial in normalizing wall tension and maintaining cardiac output, persistent hypertrophy can lead to a decline in cardiac function, ultimately resulting in HF (Nakamura and Sadoshima, 2018; Shimizu and Minamino, 2016). Pathological cardiac hypertrophy is recognized as a precursor and risk factor for HF. Therefore, timely prevention of hypertrophy has the potential to slow the transition to HF and improve clinical outcomes. In recent years, significant progress has been made in understanding the molecular mechanisms underlying cardiac hypertrophy. Currently, miRNAs are recognized as a major class of epigenetic regulators that exert a vital influence on the progression of cardiac hypertrophy (Ding et al., 2021).

Elevated levels of miRNA-212 in cardiomyocytes have been shown to induce hypertrophy, while reducing miR-212 can partially reverse this cellular hypertrophy. Additionally, miR-212 targets the rat transcription factor 7-like protein 2 (TCF7L2) and suppresses its expression, potentially promoting cardiomyocyte hypertrophy via this pathway (Yuan and Yuan, 2022). Furthermore, miR-214 induces mitochondrial dysfunction by targeting Sirtuin 3 (SIRT3) and is involved in myocardial hypertrophy incited by angiotensin II (Ang II) in mice. This suggests miR-214 may serve as a viable target for developing interventions aimed at treating cardiac hypertrophy (Ding et al., 2021).

Recently, miR-27a-3p has gained recognition for its significant role in cardiovascular diseases. Research indicates that miR-27a-3p can alleviate damage to heart muscle cells caused by both hypoxia/reoxygenation and exposure to lipopolysaccharide (Lozano-Velasco et al., 2015; Zhang X. L. et al., 2019). Furthermore, it is intricately linked to the development of obesity, ventricular formation, and various cardiac functions (Sassoon et al., 2016). Cao et al. (2021) discovered that in the cardiomyocyte hypertrophy model stimulated by Ang II, both miR-27a-3p and hypertrophy-associated genes were markedly overexpressed. Inhibitors of miR-27a-3p were shown to mitigate both electrical remodeling and myocardial hypertrophy. Additionally, miR-27a-3p can directly target the 3′-UTR of the Hoxa10 gene to regulate its expression at the transcriptional level. Elevated levels of Hoxa10 have been shown to counteract the electrical remodeling effects and myocardial hypertrophy induced by Ang II in cardiac cells. Hoxa10 promotes the upregulation of the potassium channel protein Kv4.3, typically suppressed in hypertrophic cardiomyocytes. Therefore, the pathway involving miR-27a-3p, Hoxa10, and Kv4.3 represents a newly discovered regulatory mechanism crucial to myocardial cell hypertrophy triggered by Ang II, providing a novel target for the clinical strategies aimed at preventing and treating cardiac hypertrophy and HF.

Studies have shown that the NFATc3 (NFAT4)-dependent pathway of the activated T-cell nuclear factor (NFAT) subtype is related to the progression of an enlarged heart muscle (Chao et al., 2019). NFATc3 activates the expression of miR-153–3p, while Suppressing NFATc3 leads to a reduction in miR-153–3p levels and fosters both mitochondrial division and cardiomyocyte enlargement by impeding the translation of the mitochondrial outer membrane protein (Mfn1) (Wang et al., 2020). During Ang II-induced HF, heat shock factor 2 (HSF2) activates the signaling pathway of the insulin-like growth factor II receptor (IGF-IIR), inducing cardiac hypertrophy. In spontaneously hypertensive rats (SHR), HSF2 expression is predominantly regulated by miR-18 and significantly diminished upon the activation of the p53 protein in cardiac tissue. Meanwhile, the absence of miR-18 in cardiac tissue significantly hampers heart performance, primarily through mechanism involving IGF-IIR-induced cardiac enlargement. When miR-18 is overexpressed in cardiomyocytes, it can protect against cardiac hypertrophy and preserve cardiac function. This experimental evidence suggests that miR-18 may serve as a treatment target to regulate cardiac function and alleviate cardiomyopathy during hypertension-induced HF (Huang et al., 2017). Furthermore, a heart-related circular RNA (HRCR) shields the myocardium against pathological hypertrophy and HF by engaging with miR-223 while boosting the expression of the heart-specific anti-apoptotic protein (ARC). this finding reveals a new regulatory pathway involving ARC, miR-223, and HRCR (Wang et al., 2016).

### 2.5 MiRNA regulates myocardial inflammatory response

Myocarditis presents histologically and clinically as diverse pathological immunological responses within cardiac tissue, although the etiology and pathogenesis remain unclear (Sagar et al., 2012). It is characterized by an absence of definitive diagnostic approaches and a lack of effective therapeutic options (Heymans et al., 2016). When certain factors—such as infectious pathogens, toxins, drugs, and autoimmune diseases—cause myocarditis, the inflammation may subside on its own or further evolve into dilated cardiomyopathy, potentially leading to acute HF (Caforio et al., 2015). Recent investigations have demonstrated that miRNAs play a role in both the causation and progression of myocarditis (Zhou et al., 2018). In recent years, advancements in molecular technology have allowed for a detailed analysis of the miRNA profile in myocarditis, revealing dysregulated miRNAs along with their corresponding mRNA and protein targets in both cardiac tissue samples (intracellular miRNA) and systemic fluids (known as circulating miRNA) (Fung et al., 2016). Dysregulated miRNAs in myocarditis exhibit phase-dependent changes closely related to cardiac function, arrhythmias, cardiomyocyte destruction, fibrosis, immune status, viral infection, and disease outcomes. Therefore, miRNAs represent potential targets for the diagnosis and treatment of myocarditis.

Heart macrophage infiltration is enhanced by cardiac extracellular vesicles (IR-EV), with miRNA-155–5p encapsulated within these IR-EVs potentially acting as an effector. IR-EVs transport miR-155–5p into macrophages, amplifying inflammatory responses by activating the JAK2/STAT1 signaling pathway. This interaction not only fosters localized inflammation in cardiac tissue but also triggers a systemic inflammatory response in distant organs. Therefore, Manipulating the IR-EVs-miR-155-5p-M1 signaling pathway through targeted offers potential for reducing inflammation and protecting the cardiovascular system(Ge et al., 2021). MiR-330–5p shows distinct expression patterns in both the brain and myocardial ischemia-reperfusion (I/R) injury, suggesting its potential as a biomarker for I/R-associated diseases(Wang L. et al., 2021). The inflammatory response triggered by I/R injury is mediated by the NOD-like receptor protein-3 (NLRP3) inflammasome signaling pathway. Evidence suggests miR-330–5p can mitigate myocardial I/R injury and inflammation by inhibiting the activation of this pathway (Zuo et al., 2021). MiR-532–3p plays a regulatory role in metabolic disorders like inflammation and obesity, and contributes to atherosclerotic thrombosis, which can lead to HF(Bauersachs et al., 2019; Huang et al., 2020). Long-chain non-coding RNA (lncRNA) metastasis-associated lung adenocarcinoma transcript 1 (MALAT1) competes with miR-532–3p, upregulating low-density lipoprotein receptor (LDLR) protein and worsening HF pathology. Suppression of MALAT1 can alleviate myocardial injury and inflammation in HF patients, while inhibiting miR-532–3p may reduce MALAT1’s protective effect on H9C2 cardiomyocyte injury. Additionally, miR-145–5p is a key miRNA involved in inflammation regulation, and taurine-upregulated gene 1 (TUG1) is a non-coding RNA linked to inflammation. MiR-145–5p reduces hypoxia-induced inflammatory in cardiac cells by directly regulating lncRNA TUG1 (Yuan et al., 2017). In CHF patients, miR-145–5p is downregulated, while TUG1 is significantly upregulated. Moreover, miR-145–5p and TUG1 closely linked to inflammatory markers such as TNF-α, IL-6, and CRP, indicating that the TUG1/miR-145–5p interaction is closely related to CHF inflammation and progression. This interaction may offer novel therapeutic targets for CHF treatment(Zhu et al., 2021).

## 3 The regulatory effect of CHM on the function of miRNAs in HF

Currently, therapeutic approaches based on miRNA have garnered significant attention in preclinical research. Numerous studies have shown that CHM compound formulas and their active ingredients can regulate the expression of miRNAs through various mechanisms (Li et al., 2021). For instance, commonly used CHM compound formulas, including *Huiyangjiuji* decoction (HYJJ), Fufang Zhenzhu Tiaozhi (FTZ), and Lingguizhugan decoction (LGZG) have been confirmed to regulate the expression of miRNAs associated with cardiovascular diseases (as shown in Table 2). Additionally, certain active ingredients of CHM, including Tan IIA, LBP and ginsenosides, have demonstrated potential therapeutic effects through the regulation of miRNA expression (as shown in Table 3). These CHMs and their active components enhance cardiovascular function through various pathways, demonstrating significant efficacy in treating HF. This affirmation not only underscores the therapeutic potential of CHM in addressing HF but also offers new perspectives and possibilities for miRNA-based therapeutic approaches.

**TABLE 2:** CHM compound treats HF by regulating miRNA.

CHM compound	Cellular or animal models	miRNAs	miRNA targets	Mechanism of action	References
HYJJ decoction	CHF induced by doxorubicin (DOX) in rats	511 differentially expressed miRNAs upregulated and downregulated	—	Inhibiting cardiomyocyte apoptosis and alleviate myocardial injury	Zhang et al. (2023b)
FTZ	A cardiac hypertrophy mouse model was established using TAC AngII-stimulated cardiomyocyte hypertrophy model	miR-214↓	SIRT3	Inhibiting hypertrophy of myocardial cells	Zhang et al. (2022a)
YQFM injection	CHF rat model was created following ligation of the left anterior descending coronary artery AngII-induced hypertrophic H9c2 cardiomyocytes; t-BHP-induced H9c2 cardiomyocyte apoptosis model	miR-219a-2-3p↓、miR-466c-5p↓、miR-702–5p↓、 miR-21–3p↑、miR-216b-5p↑、miR-381–3p↑、miR-542–3p↑	—	Inhibiting cardiomyocyte hypertrophy and apoptosis	Zhao et al. (2018)
LSZ capsule	Ang II-induced cardiac hypertrophy and fibrosis in rats Ang II-induced primary cardiomyocyte and primary cardiac fibroblast models	miR-150–5p↑	MMP14	Alleviating myocardial hypertrophy and fibrosis	Gu et al. (2022)
QLQX capsule	doxorubicin-induced CHF in rats	miR-345–3p↑	Smad3	Attenuating myocardial remodeling and fibrosis	Sun et al. (2020)
	Acute myocardial injury mouse model induced by permanent ligation of the LAD Primary cardiomyocyte hypertrophy model induced by PE	miR-199a-5p↓	—	Inhibiting cardiomyocyte hypertrophy	Zhang et al. (2016)
	Left coronary artery ligation-induced HF model in rats after myocardial infarction	miR133a↑	GRP78	Inhibiting excessive endoplasmic reticulum stress and reduce cardiomyocyte apoptosis	Ji et al. (2024)
LGZG decoction	Doxorubicin-induced heart failure in rats	miR-24↓	Junctophilin-2	Improving TT-SR microstructural remodeling and attenuating DOX-induced HF	Li et al. (2019c)
Shenfu injection	Rats with myocardial hypertrophy induced by abdominal aortic constriction Myocardial hypertrophy induced by phenylephrine	miR-19a-3p↑	MEF2A	Reducing myocardial hypertrophy and inhibiting apoptosis	Mao et al. (2018)

**TABLE 3:** CHM ingredient treats HF by regulating miRNA.

CHM ingredient	Cellular or animal models	miRNAs	miRNA targets	Mechanism of action	References
Tan IIA	The rat heart cell H9c2 was treated by either H2O2 or doxorubicin (DOX)	miR-133↑	Caspase-9	Inhibiting myocardial apoptosis	Song et al. (2017)
LBP	Tg mouse line for cardiac-specific overexpression of miR-1 Neonatal rat ventricular cardiomyocytes treated with miR-1 overexpression	miR-1↓	CaM、cMLCK	The protective effect on cardiac conduction function	Zhang et al. (2018b)
Ginsenosides	Rat model of myocardial ischemia/reperfusion injury	miR-144–3p↓	SLC7A11	Attenuating myocardial iron death induced by ischemia/reperfusion	Ye et al. (2023)
	Mouse model of acute myocardial infarction (AMI) and AngII-induced cardiac fibroblast (CFs) model	miR-489↑	Myd88	Inhibiting myocardial fibrosis	Sun et al. (2023)
	The left anterior descending branch-ligated HF rat model and oxygen-glucose deprivation/reoxygenation (OGD/R) H9c2 cell model	miR-216a-5p↓	Bcl2/Bax	Inhibiting myocardial cell apoptosis and oxidative stress, regulating autophagy levels	Peng et al. (2023)
PNS	Isoproterenol (ISO)-induced cardiomyocyte injury in mice	miR-29c↑	TGF-β	Reducing myocardial injury and fibrosis	Liu et al. (2017)
TGP	Hypoxia/reoxygenation (H/R) induced necroptosis in HL-1 cardiomyocytes	miR-181a-5p↓	ADCY1	Inhibiting myocardial cell pyroptosis	Yan and Huang (2021)

### 3.1 CHM compound treats HF by regulating miRNA

#### 3.1.1 *Huiyangjiuji* decoction

The traditional Chinese medicinal formula HYJJ decoction has been shown to be effective in treating acute gastroenteritis and is also suitable for alleviating symptoms such as vomiting, diarrhea, and shock. In addressing cardiac conditions such as HF and myocardial infarction, HYJJ decoction has demonstrated therapeutic potential and can be utilized for managing various acute symptoms (Yu et al., 2021). Zhang H. et al. (2023) found that HYJJ decoction can rehabilitate cardiac performance in rats and prevent the programmed cell death of heart muscle cells in rats with CHF induced by Dox, thereby alleviating myocardial damage. Its mechanism of action may be related to the regulation of specific critical miRNAs or signaling pathways associated with CHF. In this study, differentially expressed (DE) lncRNAs were identified by comparing the expression levels of lncRNAs among the HYJJ-treated, model, and control groups. RNA sequencing and bioinformatics analyses revealed that, of the 548 DE lncRNAs, the expression levels of 511 miRNAs were significantly altered, with 90 DE miRNA genes identified in the HYJJ-treated rats. KEGG and Gene Ontology (GO) analyses indicated that the differentially expressed genes were primarily enriched in the adrenergic signaling pathway in cardiomyocytes, suggesting that HYJJ decoction may exert cardioprotective effects through this signaling pathway.

#### 3.1.2 Fufang Zhenzhu Tiaozhi

FTZ is well-known for its multi-target characteristics and is widely used in China to manage hyperlipidemia and metabolic syndrome (Hu et al., 2014). FTZ has demonstrated significant effects in improving cardiovascular diseases, particularly in cases of heart muscle damage in models of long-term ischemia (Song et al., 2021), acute myocardial ischemia-reperfusion (MIR) (Qiao and Xu, 2016), and HF caused by myocardial hypertrophy. In the study conducted Zhang et al. (2022a) to explore the underlying mechanism behind FTZ’s anti-hypertrophic properties, miRNAs associated with hypertrophy, such as miR-181a, miR-206, and miR-214, were identified. The findings suggested that FTZ reduced the expression of miR-214, concurrently leading to an upregulation of the intrinsic levels of SIRT3. FTZ has the potential to mitigate HF caused by pressure-induced cardiac hypertrophy by inactivating the miR-214/SIRT3 signaling pathway.

#### 3.1.3 YiQiFuMai injection

YiQiFuMai injection (YQFM), which is derived from the traditional prescription Sheng Mai San. Extensive clinical trials have demonstrated that the application of YQFM injection in the management of CHF is both effective and associated with minimal adverse effects (Li F. et al., 2019). For example, the active constituents of YQFM injection can mitigate myocardial damage caused by HF and hypoxia. The protective influence is achieved by suppressing the NF-κB signaling cascade and the production of cytokines (Zhang Y. et al., 2019). Furthermore, YQFM injection regulates the MAPK signaling pathway, thereby diminishing myocardial remodeling and HF triggered by coronary artery ligation (CAL) (Pang et al., 2017). However, limited literature exists regarding the heart-protecting benefits of YQFM injection through miRNA regulation. Zhao et al. (2018) found that YQFM injection regulates the expression of specific miRNAs. This includes the downregulation of miR-702–5p, miR-466c-5p, and miR-219a-2-3p, along with the upregulation of miR-542–3p, miR-381–3p, miR-216b-5p and miR-21–3p. This regulatory effect effectively improves the fractional shortening of the left ventricle (LVFS) and the left ventricular ejection fraction (LVEF), reduces the left ventricular size, and increase cardiac output, thereby providing protective benefits to the cardiac tissue.

#### 3.1.4 LongShengZhi capsule

LongShengZhi (LSZ) capsule is a carefully selected traditional Chinese medicine preparation, meticulously formulated from various herbal ingredients and animal medicinal materials. This complex formula endows LSZ capsule with significant therapeutic value in treating cardiovascular diseases, particularly in alleviating atherosclerosis (Ma et al., 2019), carrageenan-induced thrombosis (Li Q. et al., 2019), and doxorubicin-induced HF (Xu et al., 2020). Research has shown that LSZ capsule exerts a protective influence against cardiac hypertrophy and fibrosis induced by Ang II in rats, and this protective impact is associated with the expression levels of miR-150–5p. In the heart disease model induced by Ang II, the expression of miR-150–5p is diminished, whereas LSZ capsule is capable of preserving its expression levels. Overexpressing miR-150–5p *in vivo* alleviates cardiac fibrosis and hypertrophy caused by Ang II, and reverses the upregulation of biomarkers associated with heart disease (ANP, BNP, β-MHC). Additionally, the gene encoding matrix metalloproteinase 14 (MMP14) is regulated by miR-150–5p, establishing it as a molecular target. Its overexpression promotes remodeling of cardiac muscle and counteracts the protective effect of miR-150–5p on cardiac hypertrophy and fibrosis (Gu et al., 2022).

#### 3.1.5 Qiliqiangxin capsule

Qiliqiangxin (QLQX) capsule is a formula composed of 11 ancient Chinese medicinal herbs that have been extensively utilized in treating chronic HF and have demonstrated significant efficacy (Tang and Huang, 2013; Zou et al., 2012). Sun et al. (2020) revealed the mechanism of action of QLQX capsule, finding that QLQX capsule inhibits the TGF-β1/Smad3 signaling pathway by upregulating miR-345–3p and promotes the activation of the TGF-β3/Smad7 signaling pathway, thereby reducing myocardial remodeling and fibrosis. This discovery provides a new perspective on the use of QLQX capsule for treating cardiac fibrosis. Zhang et al. (2016) found that the elevated levels of miR-199a-5p significantly mitigate the anti-hypertrophic effect of QLQX capsule on cardiomyocytes, manifesting as increased cardiomyocyte volume, elevated hypertrophic markers, and enhanced protein synthesis. These results emphasize the critical role of miR-199a-5p in cardiac hypertrophy and suggests that the downregulation of miR-199a-5p may aid in treating cardiac hypertrophy, potentially contributing to the mechanism by which QLQX capsule exerts its cardioprotective effect. Among the most abundant miRNAs in healthy myocardial tissue, miR-133a emerges as a crucial contributor to a wide array of biological processes. These processes include those critical to heart function, such as growth, cell proliferation, and the development of distinct cell types (Puthanveetil and O'Hagan, 2022). Upregulation of miR-133a can inhibit apoptosis of cardiomyocytes triggered by hypoxic conditions in the myocardium. Research findings indicate that QLQX capsule can significantly increase the expression of miR-133a while simultaneously suppressing glucose-regulated protein 78 (GRP78) expression in the heart, and inhibiting overactivated endoplasmic reticulum stress (ERS), thereby reducing apoptosis of myocardial cells (Ji et al., 2024).

#### 3.1.6 Lingguizhugan decoction

LGZG decoction is a time-honored herbal prescription derived from the ancient medical text *Synopsis of Prescriptions for the Golden Chamber*. It is effective in regulating Spleen Yang deficiency and resolving phlegm and dampness, and is commonly used to treat diseases caused by phlegm retention, including chronic congestive HF (Liu et al., 2013). According to the principles of TCM, phlegm retention and Spleen Yang deficiency are considered important etiologies of HF; thus, LGZG decoction has a long history of use in the management of cardiovascular diseases. Li X. et al. (2019) found that LGZG decoction positively impacts the remodeling of cardiac microstructure by suppressing miR-24 expression and enhancing Junctophilin-2 (JP-2) expression, thereby alleviating Doxorubicin-induced HF. These findings reveal the potential mechanism by which LGZG decoction improves HF, providing experimental support for its use a treatment for cardiovascular diseases.

#### 3.1.7 Shenfu injection

Shenfu injection is a representative formula of traditional Chinese medicine used for replenishing Qi and warming Yang. This formula has been used in traditional practice for millennia and has demonstrated potential efficacy in the treatment of HF (Tao et al., 2023). The primary active ingredients of *Panax ginseng C.A. Meyer* [Araliaceae; Ginseng Radix] and *Aconitum carmichaeli Debeaux* [Ranunculaceae; Aconiti Carmichaeli Radix], with ginsenoside exerting a significant effect on regulating apoptosis (Peng et al., 2023). Yan et al. (2015) found that ginsenoside Rb1 can reverse the increase in miR-1, and the associated cell death caused by hypoxia and ischemia (H/I) in neonatal rat cardiomyocytes (NRCMs). Furthermore, Yan et al. (2018) discovered that Shenfu injection significantly impacts miRNAs related to cell apoptosis in HF rats (including miR-320, miR-21, miR-208, and miR-29), suggesting that Shenfu injection may balance the activation and inhibition of cell apoptosis by regulating these miRNAs, ultimately inhibiting cell apoptosis and improving cardiac function of HF rats. Mao et al. (2018) indicated that Shenfu injection can alleviate myocardial hypertrophy and reduce the mortality rate of HF and other cardiovascular conditions by modulating the concentration of miR-19a-3p, reducing the levels of myocyte enhancer factor 2A (MEF2A) mRNA and protein, and regulating proteins associated with the MEF2 signaling pathway.

### 3.2 CHM ingredient treats HF by regulating miRNA

#### 3.2.1 Tanshinone IIA

Danshen, a well-known Chinese herb, contains active ingredients extracted from its dry roots and rhizomes that have shown substantial effects on addressing cardiovascular conditions. The active ingredients of Danshen include Tan IIA, tanshinone I, salvianolic acid, and dihydrotanshinone, with Tan IIA being the predominant component (Weng et al., 2015). Clinical applications indicate that Tan IIA can treat cardiovascular issues such as HF caused by cardiac hypertrophy. Its mechanism of action include inducing apoptosis of cardiomyocytes, inhibiting the hypertrophy, alleviating cardiac fibrosis and limiting oxidative stress, thereby improving cardiac function (Chan et al., 2011; Tan et al., 2011). Feng et al. (2012) revealed that Tan IIA can diminish myocardial apoptosis in HF rats by upregulating the level of miR-133. This discovery illustrates how Tan IIA protects the myocardium at the molecular level. Song et al. (2017) further found that Tan IIA can markedly reverse the downregulation of miR-133 under adverse conditions, inhibit the expression of Caspase-9 along with associated apoptotic mediators, and effectively reduce myocardial apoptosis by suppressing the Caspase-9 signaling pathway while promoting the synthesis of miR-133, aiding in the repair of myocardial injury. Additionally, Gu et al. (2016) found that Tan IIA can protect H9C2 heart cells from cell death induced by oxidative stress. The involvement of miR-133 and Akt pathways highlights the role of Tan IIA as a natural cardioprotective agent, showcasing its significant potential.

#### 3.2.2 *Lycium barbarum* polysaccharide

*Lycium barbarum* L., a prominent traditional Chinese herb and functional food, not only has a rich historical background in China but is also gaining popularity in North America and Europe. It is believed to nourish the liver, kidneys, and eyes while offering various health benefits (Yang et al., 2017). LBP are significant bioactive ingredients derived from *lycium barbarum* L., accounting for up to 40% of the dry weight of the fruit (Li et al., 2011). LBP have been scientifically proven possess various pharmacological and biological functions, including but not limited to antioxidant properties (Varoni et al., 2017), immune regulation (Bo et al., 2016), anti-tumor activity (Zhang et al., 2017), anti-aging effects(Yi et al., 2013), neuroprotection (Li et al., 2011), blood sugar and lipid reduction (Xiao et al., 2014), and promotion of male fertility (Qian and Yu, 2016). These functions demonstrate the broad application potential of LBP in preventing and treating various cardiovascular illnesses. Notably, LBP exhibit cardioprotective effects. Research has shown that LBP can reduce myocardial apoptosis and injury during I/R in rat hearts, thereby helping to prevent cardiovascular diseases. Zhang R. et al. (2018) found that LBP can restore cardiac function in a miR-1 transgenic mouse model resulting from the overexpression of miR-1. The underlying mechanism may involve LBP downregulating miR-1 expression, thereby reversing the inhibition of target proteins related to myocardial contractility induced by miR-1. This finding strongly suggests that LBP have protective effects on cardiac conduction function, providing a scientific basis for their use in treating heart conditions.

#### 3.2.3 Ginsenosides

Ginseng (Panax ginseng C.A. Meyer), a well-known traditional Chinese herb, has been utilized since ancient times. Ginsenosides, the main active ingredient, possess a broad spectrum of pharmacological properties, including alleviation of neuroinflammation (Cai and Yang, 2016), inhibition of vascular smooth muscle cell proliferation (Gao et al., 2019), the reduction of oxidative stress and inflammation (Lee et al., 2020). Importantly, ginsenosides also exert numerous beneficial pharmacological impacts on the cardiovascular system, including angiogenic, anti-arrhythmic, and anti-ischemic effects (Cai and Yang, 2016). Research indicates that ginsenoside Re can diminish intrinsic rhythmicity and cardiac contractility, depending on dosage. This effect is attributed to its ability to enhance nitric oxide (NO) production from L-arginine and inhibit calcium (Ca2+) channel activity in endothelial cells of blood vessels (Kim et al., 2015). Additionally, ginsenosides exert heart-protective effects by regulating miRNAs. In the I/R rat model, miR-144–3p expression levels rose significantly. Excessive expression of miR-144–3p increased the ROS levels in H9C2 cells, making them sensitive to ferroptosis induced by Erastin, a ferroptosis activator. Ginsenoside Re can suppress miR-144–3p expression, leading to elevated levels of its target protein, cystine/glutamate antiporter SLC7A11, thereby inhibiting myocardial cell ferroptosis and alleviating cardiac damage caused by I/R (Ye et al., 2023). A study identified miR-489 as a key factor in myocardial cell fibrosis. It can directly target Myd88 and influence the processes of cardiac hypertrophy and fibrosis, which are regulated through the NF-κB signaling pathway. Ginsenoside Re can elevate miR-489 expression and suppress activation of the Myd88/NF-κB signaling pathway, thereby inhibiting myocardial fibrosis development (Sun et al., 2023). Another study found that ginsenoside Rb2 can reduce HF-induced myocardial remodeling, maintain cardiomyocyte morphology integrity, and restore cellular vitality (Peng et al., 2023). In this research, rats with HF and myocardial cells exposed to oxygen-glucose deprivation followed by reperfusion (OGD/r) exhibited a significant increase in miR-216a-5p expression. Ginsenoside Rb2 intervention could reduce miR-216a-5p activity and upregulate Beclin1, LC3B II/I, and Bcl2 expression in myocardial cells while downregulating Caspase-3, Bax, and p62 levels. Conversely, upregulation of miR-216a-5p enhances apoptosis and induce oxidative stress in cardiomyocytes. Concurrently, it suppressed autophagy, thereby neutralizing the beneficial effects of ginsenoside Rb2 on HF in both *in vitro* and *in vivo* settings.

#### 3.2.4 *Panax notoginseng* saponins

Sanqi, also known as *P. notoginseng*, is an herbal medicine with a rich history usage. The primary bioactive components of *P. notoginseng* are its saponins, collectively referred to as PNS. This complex chemical composition is rich in various dammarane-type saponins (Xu et al., 2019). Extensive research has demonstrated the broad applications of PNS in both medical studies and clinical practice, including diabetes mellitus, atherosclerosis, malignancies, and cardiovascular disorders (Uzayisenga et al., 2014). The cardioprotective mechanism of PNS is linked to their ability to modulate gene expression via miRNAs. miR-29 functions as an anti-fibrotic agent that interacts with the coding sequence of TGF-β. This interaction effectively suppresses the TGF-β signaling cascade, thereby alleviating myocardial fibrosis. In mouse cardiac tissue induced by ISOP, a marked decrease in the expression levels of miR-29 is observed. Treatment with PNS enhance cardiac expression levels of miR-29, simultaneously reduces the activity of the TGF-β signaling pathway, and reverses the myocardial damage and fibrosis induced by ISOP (Liu et al., 2017).

#### 3.2.5 Total glucosides of paeony

Derived from the desiccated rhizomes of the *Paeonia lactiflora* Pall, TGP constitute a key component of this Chinese herb. This compound exhibits a range of pharmacological effects, including immunomodulatory capabilities, as well as antioxidant and anti-inflammatory properties, demonstrating significant potential in addressing various cardiovascular conditions (Wei et al., 2024). Evidence indicates that TGP significantly reduces inflammation in patients with stable CHF by suppressing proinflammatory cytokine production, thereby delaying disease progression (Lin et al., 2020). In cardiomyocytes, TGP mitigates apoptosis and oxidative stress triggered by I/R by suppressing the PI3K/Akt axis (Shen et al., 2018). Furthermore, TGP protects cardiomyocytes from damage by regulating miRNA. Evidence suggests that in H/R cardiomyocytes, upregulation of miR-181a-5p expression is observed. In addition to suppressing miR-181a-5p expression, TGP also regulates the adenosine cyclase 1 (ADCY1) gene by directly targeting this miRNA. When miR-181a-5p is overexpressed or an ADCY1 inhibitor is applied, the suppressive effect of TGP on H/R-triggered pyroptosis in cardiomyocytes is diminished or reversed (Yan and Huang, 2021).

## 4 Discussion and perspective

HF represents a serious clinical condition defined by the heart’s diminished capacity to efficiently circulate blood, thereby failing to adequately meet the body’s needs. HF is typically triggered by various cardiovascular conditions, including hypertension, coronary heart disease (CHD), and different types of cardiomyopathies (Lehnart, 2013; Savarese et al., 2023). Treatment of HF typically involves the use of beta-adrenergic receptor blockers, diuretics, and angiotensin-converting enzyme inhibitors (ACEIs). These medications can alleviate symptoms, prevent disease progression, and improve both the quality of life and survival odds for patients (Al-Mohammad and Mant, 2011). However, prolonged use of such pharmaceutical agents may lead to severe consequences, such as hypotension, fluid depletion, and electrolyte imbalances, thereby negatively impacting the overall health of patients (Gupta et al., 2014; Yancy et al., 2013). Modern medical advances have significantly contributed to the reduction of both the incidence and mortality rates associated with HF (Bozkurt and Mann, 2013); however, the economic and social burden of HF continues to rise. Therefore, exploring the pathogenesis of HF and developing new effective drugs with fewer side effects will be crucial for its prevention and treatment. CHM is emerging as a novel approach to the prevention and treatment of diseases due to its multi-target mechanisms of action and fewer side effects (Wang et al., 2017). Clinical assessments and laboratory investigations have demonstrated that CHM and its derived compounds have the potential to serve as effective treatments for HF (Li and Xu, 2013).

Studies have demonstrated that CHM can regulate pathophysiological processes in HF through a complex array of mechanisms. This includes effects on relevant signaling pathways, non-coding microRNAs, and stem cells. Through these regulatory effects, CHM exerts multiple biological impacts, including inhibition of apoptosis, antioxidant effects, anti-cardiac myocyte hypertrophy, anti-fibrotic effects, and anti-inflammatory effects, thereby demonstrating its unique advantages in the treatment of HF (Hao et al., 2017). CHM typically contain a variety of bioactive components that can act on multiple molecular targets, thereby modulating pathological processes at various levels. The multi-target action characteristic of CHM enables intervention in the disease process from various angles when treating HF, potentially leading to more comprehensive therapeutic effects compared to traditional single-target drug therapies. Additionally, CHM typically has fewer side effects, which is particularly important for patients with chronic diseases requiring long-term treatment. Moreover, the efficacy of CHM has been shown to be significant in many cases, providing tangible clinical benefits to patients (Wu et al., 2022). For instance, the QLQX Capsule improved the prognosis of patients with HF in a double-blind clinical trial, with results published in the prestigious international journal *Nature Medicine*, highlighting the significant value and potential of Chinese medicine in HF treatment (Cheang et al., 2024). However, despite these positive findings, clinical trials of CHM for the treatment of HF remain relatively limited, and the quality of these studies varies. This situation may be attributed to the inherent complexity of CHM research, challenges in clinical trial design, and the lack of standardized assessments of CHM efficacy, among other factors. In light of this, there is an urgent need for broader, multi-institutional, high-caliber controlled clinical studies, along with practical research. Simultaneously, to provide empirical guidance for the clinical utilization of CHM, it is imperative to conduct a more in-depth exploration of the specific targets and associated signaling pathways of CHM and its diverse bioactive constituents. Furthermore, a multidisciplinary approach is required, combining traditional Chinese medical theory with contemporary biomedical techniques to design more rigorous and scientifically sound clinical trials, thereby promoting the use of CHM for the treatment of HF.

MiRNAs influence a multitude of biological processes by modulating gene expression, including the onset and progression of heart diseases. In recent years, a deeper understanding of miRNAs in cardiovascular diseases has led to a growing body of research focusing on the effects of CHM on miRNAs, opening novel avenues and methodologies for addressing HF (Wu et al., 2023). For example, certain compounds and active components of CHM, such as HYJJ decoction, YQFM Injection, QLQX, Tan IIA, and LBP, are reported to exert cardioprotective effects through the modulation of specific miRNAs. Although existing research has progressed in revealing the regulatory effects of miRNAs in CHM on cardiovascular diseases, providing initial insights into the mechanisms of action, overall knowledge of the complex interactions and specific mechanisms of these miRNA molecules under different disease conditions remains limited. Additionally, since CHM consists of a variety of complex ingredients, focusing solely on a single miRNA or molecular target may not fully reveal its therapeutic potential. Therefore, it is crucial to delve into the miRNA network and its complex molecular mechanisms underlying the action of CHM, and focusing on specific miRNAs as targets could emerge as a promising avenue for addressing HF.

In summary, miRNAs exert a pivotal regulatory influence on the onset and progression of HF, and CHM has demonstrated significant therapeutic potential by regulating miRNA expression. Future research should further investigate the specific mechanisms of interaction between CHM and miRNA to develop new treatment strategies based on miRNA regulation. This will not only enrich treatment methods for HF but also provide new perspectives for personalized therapy.

## Glossary

HF: Heart Failure
CHM: Chinese Herbal Medicine
miRNA: MicroRNA
3′-UTR: 3′-Untranslated Region
TCM: Traditional Chinese Medicine
Tan IIA: Tanshinone IIA
LBP: Lycium Barbarum Polysaccharide
PNS: Panax Notoginseng Saponins
TGP: Total Glucosides of Paeony
Dox: Doxorubicin
ATG: Autophagy-related gene
APF: Autophagy-promoting factor
mTOR: Mechanistic Target of Rapamycin
CDK: Cyclin-Dependent Kinase
ATP: Adenosine Triphosphate
RISC: RNA-Induced Silencing Complex
MEG3: Maternal Expression Gene 3
ECM: Extracellular Matrix
AMPK: AMP-activated Protein Kinase
HNF-4α: Hepatocyte Nuclear Factor 4 Alpha
TGF-β1: Transforming Growth Factor Beta 1
Peli1: E3 Ubiquitin Ligase Peli1
TNFR: Tumor Necrosis Factor Receptor
TLR: Toll-like Receptor
IL-1R: Interleukin-1 Receptor
CHF: Congestive Heart Failure
HSF2: Heat Shock Factor 2
NFAT: Nuclear Factor of Activated T-cells
TRPM7: Transient Receptor Potential Melastatin 7
lncRNA: Long Non-coding RNA
LDLR: Low-Density Lipoprotein Receptor
TUG1: Taurine-upregulated Gene 1
I/R: Ischemia/Reperfusion
JAK2: Janus Kinase 2
STAT1: Signal Transducer and Activator of Transcription 1
HYJJ: Huiyangjiuji Decoction
FTZ: Fufang Zhenzhu Tiaozhi
LGZG: Lingguizhugan Decoction
YQFM: YiQiFuMai Injection
NF-κB: Nuclear Factor kappa-light-chain-enhancer of activated B cells
MAPK: Mitogen-Activated Protein Kinase
CAL: Coronary Artery Ligation
DE: Differentially Expressed
SIRT3: Sirtuin 3
Smad3: Sma- and Mad-Related Protein 3
LVEF: Left Ventricular Ejection Fraction
LVFS: Left Ventricular Fraction Shortening
MMP14: Matrix Metalloproteinase 14
GRP78: Glucose-Regulated Protein 78
JP-2: Junctophilin-2
H/I: Hypoxia and Ischemia
MEF2A: Myocyte Enhancer Factor 2A
NO: Nitric Oxide
Ca2+: Calcium
ROS: Reactive Oxygen Species
ADCY1: Adenylate Cyclase 1
PI3K: Phosphoinositide 3-Kinase
Akt: Protein Kinase B
ISOP: Isoproterenol
H/R: Hypoxia/Reperfusion
