# Supplementary material for: Exploring the therapeutic mechanisms of heart failure with Chinese herbal medicine: a focus on miRNA-mediated regulation
Source: Front Pharmacol. 2024 Nov 5;15:1475975. doi: 10.3389/fphar.2024.1475975 (PMC11573571; doi:10.3389/fphar.2024.1475975)
Supplement: Supplementary file 1 [file DataSheet1.PDF]

Supplementary material Table 1. Specific names of Chinese herbal medicine compositions

CHM compound	Composition/Ingredients
HYJJ decoction	1. *Aconitum carmichaelii* Debx. [Ranunculaceae; Aconiti carmichaelii radix]<br>2. *Zingiber officinale* Roscoe [Zingiberaceae; Zingiberis rhizoma]<br>3. *Panax ginseng* C.A. Meyer [Araliaceae; Panacis ginseng radix]<br>4. *Glycyrrhiza uralensis* Fisch. [Fabaceae; Glycyrrhizae radix et rhizoma]<br>5. *Atractylodes macrocephala* Koidz. [Asteraceae; Atractylodis macrocephalae rhizoma]<br>6. *Cinnamomum cassia* Presl [Lauraceae; Cinnamomi cortex]<br>7. *Citrus reticulata* Blanco [Rutaceae; Citri reticulatae pericarpium]<br>8. *Schisandra chinensis* (Turcz.) Baill. [Schisandraceae; Schisandrae chinensis fructus]<br>9. *Poria cocos* (Schw.) Wolf [Polyporaceae; Poria]<br>10.*Pinellia ternata* (Thunb.) Breit. [Araceae; Pinelliae rhizoma]
FTZ	1. *Citrus medica* L. var. sarcodactylis (H. S. Cheng) H. S. Cheng [Rutaceae; Citri sarcodactylis fructus]<br>2. *Ligustrum lucidum* W.T. Aiton [Oleaceae; Ligustri lucidi fructus]<br>3. *Salvia miltiorrhiza* Bunge [Lamiaceae; Salviae miltiorrhizae radix et rhizoma]<br>4. *Panax notoginseng* (Burkill) F.H. Chen [Araliaceae; Notoginseng radix et rhizoma]<br>5. *Coptis chinensis* Franch. [Ranunculaceae; Coptidis rhizoma]<br>6. *Atractylodes macrocephala* Koidz. [Asteraceae; Atractylodis macrocephalae rhizoma]<br>7. *Cirsium japonicum* (Thunb.) Matsum. [Asteraceae; Cirsii japonici herba et radix]<br>8. *Eucommia ulmoides* Oliv. [Eucommiaceae; Eucommiae cortex]
YQFM injection	1. *Panax ginseng* C.A. Mey. [Araliaceae; Ginseng Radix]<br>2. *Ophiopogon japonicus* (Thunb.) Ker Gawl [Liliaceae; Ophiopogonis Radix]<br>3. *Schisandra chinensis* (Turcz.) Baill [Schisandraceae; Schisandrae Fructus]
LSZ capsule	1. *Astragalus mongholicus* Bunge [Fabaceae; Astragali mongholici radix]<br>2. *Hirudo nipponica* Whitman [Hirudinidae; Hirudo]<br>3. *Conioselinum anthriscoides* 'Chuanxiong' [Apiaceae; Conioselini anthriscoidis radix]<br>4. *Angelica sinensis* (Oliv.) Diels [Apiaceae; Angelicae sinensis radix]<br>5. *Carthamus tinctorius* L. [Asteraceae; Carthami flos]<br>6. *Prunus persica* (L.) Batsch [Rosaceae; Persicae pericarpium]

	7. *Paeonia lactiflora* Pall. [Paeoniaceae; Paeoniae radix]
	8. *Dolomiaea costus* (Falc.) Kasana & A.K.Pandey [Asteraceae; Saussureae radix]
	9. *Acorus gramineus* Aiton [Acoraceae; Acori graminei rhizoma]
	10. *Pheretima aspergillum* (E. Perrier) [Lumbricidae; Pheretimae]
	11. *Taxillus chinensis* (DC.) Danser [Loranthaceae; Taxilli herba]
	12. *Eleutherococcus senticosus* (Rupr. & Maxim.) Maxim. [Araliaceae; Eleutherococci senticosi radix]
QLQX capsule	1. *Astragalus membranaceus* (Fisch.) Bunge [Fabaceae; Astragali radix]
	2. *Aconitum carmichaelii* Debeaux [Ranunculaceae; Aconiti lateralis radix preparata]
	3. *Panax ginseng* C.A. Meyer [Araliaceae; Ginseng radix et rhizoma]
	4. *Salvia miltiorrhiza* Bunge [Lamiaceae; Salviae miltiorrhizae radix et rhizoma]
	5. *Alisma plantago-aquatica* subsp. orientale (Sam.) Sam. [Alismataceae; Alismatis rhizoma]
	6. *Descurainia sophia* (L.) Webb ex Prantl [Brassicaceae; Descuraunia semen]
	7. *Cinnamomum cassia* (L.) J. Presl [Lauraceae; Cinnamomi ramulus]
	8. *Carthamus tinctorius* L. [Asteraceae; Carthami flos]
	9. *Periploca sepium* Bunge [Asclepiadaceae; Periplocae cortex]
	10. *Polygonatum odoratum* (Mill.) Druce [Convallariaceae; Polygonati odorati rhizoma]
	11. *Citrus reticulata* Blanco [Rutaceae; Citri reticulatae pericarpium]
LGZG decoction	1. *Smilax glabra* Roxb. [Smilacaceae; Smilacis glabrae rhizoma]
	2. *Neolitsea cassia* (L.) Kosterm. [Lauraceae; Cinnamomi ramulus]
	3. *Atractylodes macrocephala* Koidz. [Asteraceae; Atractylodis macrocephalae rhizoma]
	4. *Glycyrrhiza glabra* L. [Fabaceae; Glycyrrhizae radix et rhizoma]
Shenfu injection	1. *Panax ginseng C.A. Meyer* [Araliaceae; Ginseng Radix]
	2. *Aconitum carmichaeli Debeaux* [Ranunculaceae; Aconiti Carmichaeli Radix]
